# Travel datasets for analysing the electric vehicle charging demand in a university campus

**DOI:** 10.1016/j.dib.2024.110335

**Published:** 2024-03-16

**Authors:** Yan Wu, Syed Mahfuzul Aziz, Mohammed H. Haque

**Affiliations:** UniSA STEM, University of South Australia, Mawson Lakes, SA, Australia

**Keywords:** Electric vehicle (EV), Probability distribution, Vehicle travel data, Campus parking data, Workplace charging demand, Energy management, Cost optimisation

## Abstract

This article presents travel datasets of privately used vehicles for the determination of the daily charging demand of electric vehicles (EV) at a university campus and to analyse strategies to minimise the annual energy cost. The datasets have been used in the primary research article published in the *Renewable Energy* journal [1]. The original raw data of vehicle usage is sourced from the Victorian Integrated Survey of Travel & Activity (VISTA) [2], which is an ongoing survey led by the Department of Transport and Planning of the Victorian State Government. Since 2012, data collection has been evenly distributed across each year, with 32,000 households and 82,000 individuals participating in the ongoing survey. The raw dataset is filtered and processed to obtain the daily travel distance and workplace arrival–departure times of privately used vehicles. Probability distributions and cumulative distributions of the daily travel distance and workplace arrival–departure times respectively are extracted. Using these distributions, the year-round travel data (daily travel distance and workplace arrival–departure times) is created for the desired number of EVs individually. These are used to generate the daily EV charging demand profile at the workplace so that appropriate charging strategies and cost optimisation methods can be tested. The experimental methods used to obtain the required data, from downloading the raw dataset to creating the individual EV's travel data are described in this paper.

Specifications Table

**Table:** 

Subject	Electrical Engineering
Specific subject area	Probability distribution of vehicle travel data, workplace charging of electric vehicle (EV), charging demand, energy cost optimisation
Data format	Raw, Filtered, Processed, Created
Type of data	Excel files, Tables, Figures
Data collection	Raw travel dataset “*T_VISTA1218_V1.csv*” is obtained by decompressing the dataset downloaded from the Victorian Integrated Survey of Travel & Activity [2].
Data source location	Victorian Integrated Survey of Travel & Activity [2]. Institution: Victoria State Government – Department of Transport City/Town/Region: Victoria Country: Australia
Data accessibility	Repository name: MendeleyData Data identification number: 10.17632/8kpvtwxxtx.3 Direct URL to data: https://data.mendeley.com/datasets/8kpvtwxxtx/3
Related research article	Y. Wu, S. M. Aziz, M. H. Haque, Techno-economic modelling for energy cost minimisation of a university campus to support electric vehicle charging with photovoltaic capacity optimisation, Renewable Energy. 219 (2023) 119427. https://doi.org/10.1016/j.renene.2023.119427. [1]

## 1 Value of the Data

• With the expected proliferation of electric vehicles [3,4], it is important for workplaces to plan for the infrastructure required to support on-site EV charging demand while also investigate cost optimisation strategies [5], [6], [7], [8]. To determine the charging demand caused by the EVs, credible datasets are needed on the cumulative distributions of the vehicles arriving at the workplace, departing the workplace and parked at the workplace. This paper presents such datasets based on the processing of the raw dataset sourced from the VISTA website.
• Probability distributions of daily travel distance, workplace arrival and departure times of vehicles are obtained after filtering and processing the raw VISTA dataset. These distributions are useful to create, for any number of EVs, the daily travel distance and workplace arrival–departure times of individual vehicles as well as cumulative distributions of vehicles arriving at the workplace, departing the workplace and parked at the workplace.
• The above cumulative distributions represent credible sets of data on vehicle parking at the workplace because they were validated using year-round vehicle parking data from a real campus. The cumulative distributions and the probability distributions mentioned above are crucial to determine the EV charging load demand at the workplace (campus).
• Consequently, the EV charging demand profile, created based on the probability distributions and cumulative distributions, represent creadible EV charging load profile at the workplace. The original research article [1] utlised such charging load profile to analyse the techno-economic model for energy cost minimisation of a university campus. The dataset presented can be scaled for the number of EVs to suit the workplace and hence has wider scope. The methodology used to obtain the processed and created travel datasets can be applied to workplaces in other regions or countries because these methods are not limited to any specific region or country.
• The charging load profile created from the presented datasets can be used to optimise workplace energy costs by developing suitable EV charging strategies. The charging load profile for an EV fleet can be used to analyse the impact of workplace charging on distribution networks. The charging load profile can also be used to obtain valuable insights into workplace charging for planning purposes, for example, to determine the charging facilities required.
• Researchers, policymakers, property owners and charging station operators can utilize the presented vehicle travel patterns for planning and design purposes; while, researchers can use the datasets to reproduce the results reported in the original research article [1], compare the results with those obtained using other datasets from different regions/countries, and build on this for future research.

## 2 Data Description

The datasets provided in this paper are summarized in Table 1. These include one raw travel dataset, one filtered dataset, three processed datasets and three created datasets as described in Table 1. Brief descriptions of the contents of these datasets are given in this section. The methods used to obtain each of these datasets are given in Section 3.

**Table 1:** List of datasets available with this paper.

No.	Name	Type	Description
1	*T_VISTA1218_V1.csv* (including *VISTA - Glossary of Variables 12-18.docx*)	Raw dataset	Contains travel distance, arrival & departure times of all trips during the day for all types of commuting modes (e.g., walking, bus, private vehicle and train).

2	*VISTA PrivateVehicle.csv*	Filtered dataset	Travel distance, arrival & departure times of all trips for only privately used vehicles.

3	*VISTA DailyDistance and WorkArrDepTime.csv*	Processed datasets	The total distance travelled in a day obtained by combining all trips of the day; workplace arrival and departure times obtained by removing all destination locations except workplace.
4	*VISTA ProbabilityDistribution.csv*		The probability distribution of the daily travel distance, workplace arrival time and departure time.
5	*VISTA CumulativeDistribution.csv*		The cumulative distribution of the vehicles arriving at the workplace, departing the workplace and parked at the workplace.

6	*EV Daily D.csv*	Created datasets	The daily travel distance created individually for the designated number of EVs using the relevant probability distribution from the Processed dataset.
7	*EV Workplace Tar.csv*		The daily workplace arrival time created individually for the designated number of EVs using the relevant probability distribution from the Processed dataset.
8	*EV Workplace Tde.csv*		The daily workplace departure time created individually for the designated number of EVs using the relevant probability distribution from the Processed dataset.

### 2.1 T_VISTA1218_V1.csv (including VISTA - Glossary of Variables 12-18.docx)

The travel survey data *T_VISTA1218_V1.csv* is the raw dataset used in the original research article [1]. In the dataset, Trip refers to the uninterrupted commuting from one departure location to one arrival location, and daily travel may consist of one or more Trips. The main data include Trip ID, Time of departure and arrival, Trip location, Trip purpose, and commuting mode (whether driver or passenger in a privately used vehicle, bus, train etc.). The specific definition of each column can be found in the instruction file *VISTA - Glossary of Variables 12-18.docx*.

### 2.2 VISTA PrivateVehicle.csv

*VISTA PrivateVehicle.csv* is a filtered dataset that contains the travel survey data for all trips only for privately used vehicles. The data definition of each column is the same as that of the raw dataset.

### 2.3 VISTA DailyDistance and WorkArrDepTime.csv

*VISTA DailyDistance and WorkArrDepTime.csv* describes the daily travel pattern of privately used vehicles, including the total daily travel distance, workplace arrival time and workplace departure time. In this processed dataset, there are 27,771 data for daily travel distance, 16,223 data for workplace arrival time, and 15,731 data for workplace departure time. Note that the number of data items for daily travel distance is larger than the number of data items for either workplace arrival time or workplace departure time, because not all privately used vehicles are used for commuting to and from work. The difference between the number of data items for workplace arrival time and workplace departure time is not known; these are the numbers obtained from the original raw dataset. Table 2 provides the description of each column in the dataset.

**Table 2:** Description of dataset: VISTA DailyDistance and WorkArrDepTime.csv.

Column #	Name	Description
1	DISTANCE	Total daily travel distance (in km)
2	ARRTIME	Time of arrival at the workplace (in minutes, from midnight)
3	DEPTIME	Time of departure from the workplace (in minutes, from midnight)

### 2.4 VISTA ProbabilityDistribution.csv

*VISTA ProbabilityDistribution.csv* describes the probability distributions of daily travel distance, workplace arrival times and departure times of all privately used vehicles. Intervals of 5 km and 30 min are used to calculate the probability distributions. Table 3 provides the description of each column in the dataset.

**Table 3:** Description of dataset: *VISTA ProbabilityDistribution.csv*.

Column #	Name	Description
1	INDEX_ DISTANCE	Index of daily travel distance (5 km interval)
2	PD_DISTANCE	Probability distribution of daily travel distance
3	INDEX_TIME	Index of workplace arrival time (30 min interval, 48 time plots starting from midnight)
4	PD_ARRTIME	Probability distribution of workplace arrival time
5	INDEX_TIME	Index of workplace departure time, which is the same as the INDEX used for arrival time (30 min interval, 48 time plots from midnight)
6	PD_DEPTIME	Probability distribution of workplace departure time

### 2.5 VISTA CumulativeDistribution.csv

**Fig. 1:**
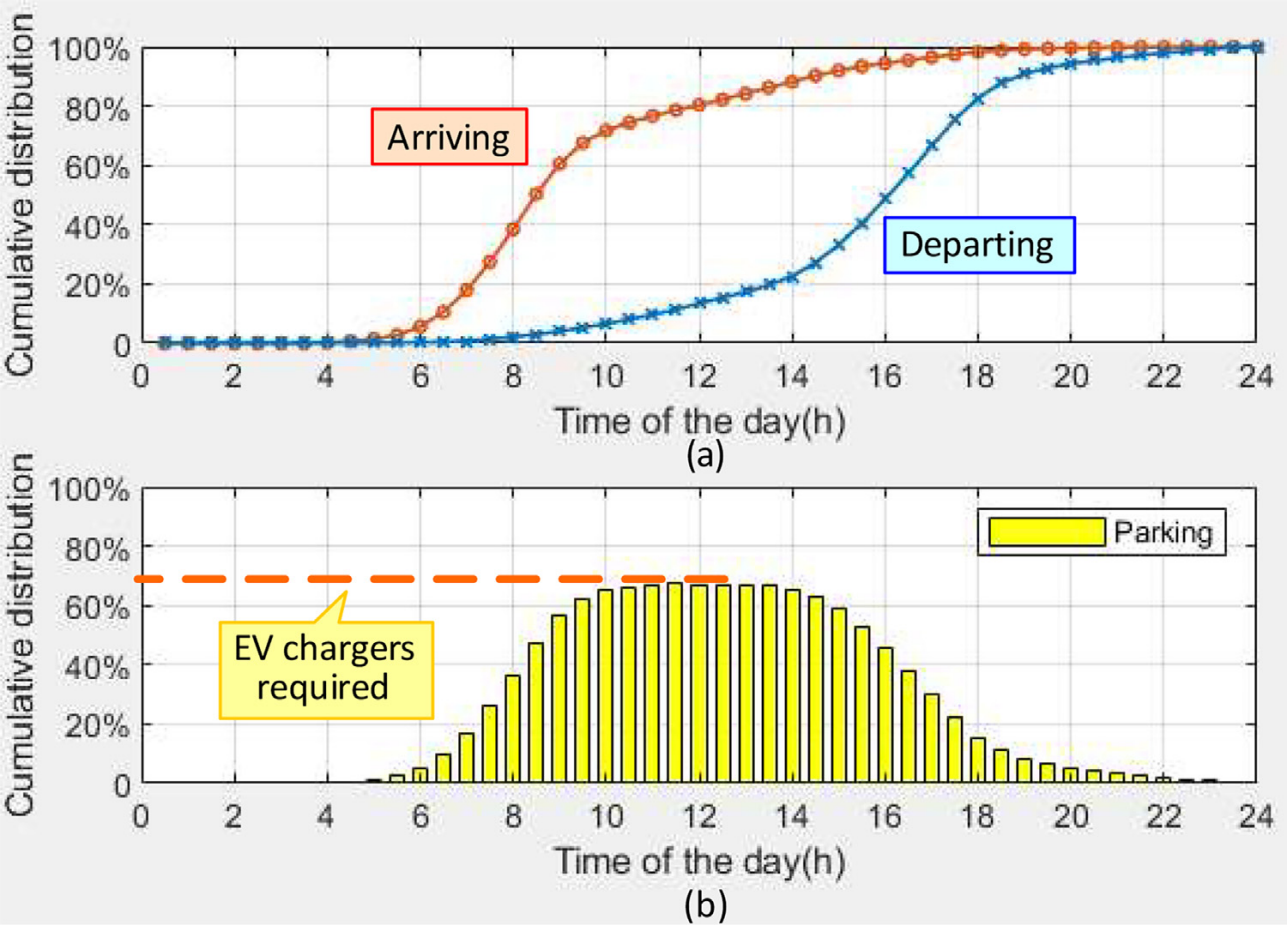
Cumulative distributions of (a) vehicles arriving and departing the workplace and (b) parking at the workplace.

*VISTA CumulativeDistribution.csv* contains the cumulative distributions of the vehicles arriving at the workplace and departing the workplace throughout the day. These two distributions are used to generate the cumulative distribution of vehicles parked at the workplace, which is also included in this dataset. Table 4 provides the description of each column in the dataset. Calculated from the probability distribution, the cumulative distribution includes 48 time plots with 30 min interval starting from midnight. Fig. 1(a) shows the cumulative distributions of the vehicles arriving at the workplace and departing the workplace. The difference between the above two distributions represents the workplace parking distribution and is shown in Fig. 1(b). It can be observed that the highest percentage of EV parking occurs around noon (approximately 69 % of the total number of EVs). Therefore, to meet the charging needs of all EV users, the number of EV chargers required at the workplace should be greater than or equal to 69 % of the total number of EVs. As stated in Sections 2.6 and 3.6, the total number of EVs considered in the original research article is 874 [1], therefore at least 603 EV chargers are required to meet the charging demand during the peak parking period.

**Table 4:** Description of dataset: *VISTA CumulativeDistribution.csv*.

Column #	Name	Description
1	INDEX_TIME	Time index of cumulative distribution (30 min interval, 48 time plots starting from midnight)
2	CD_ARRTIME	Cumulative distribution of the vehicles arriving at the workplace
3	CD_DEPTIME	Cumulative distribution of the vehicles departing the workplace
4	CD_PARK	Cumulative distribution of the vehicles parking at the workplace

**Fig. 2:**
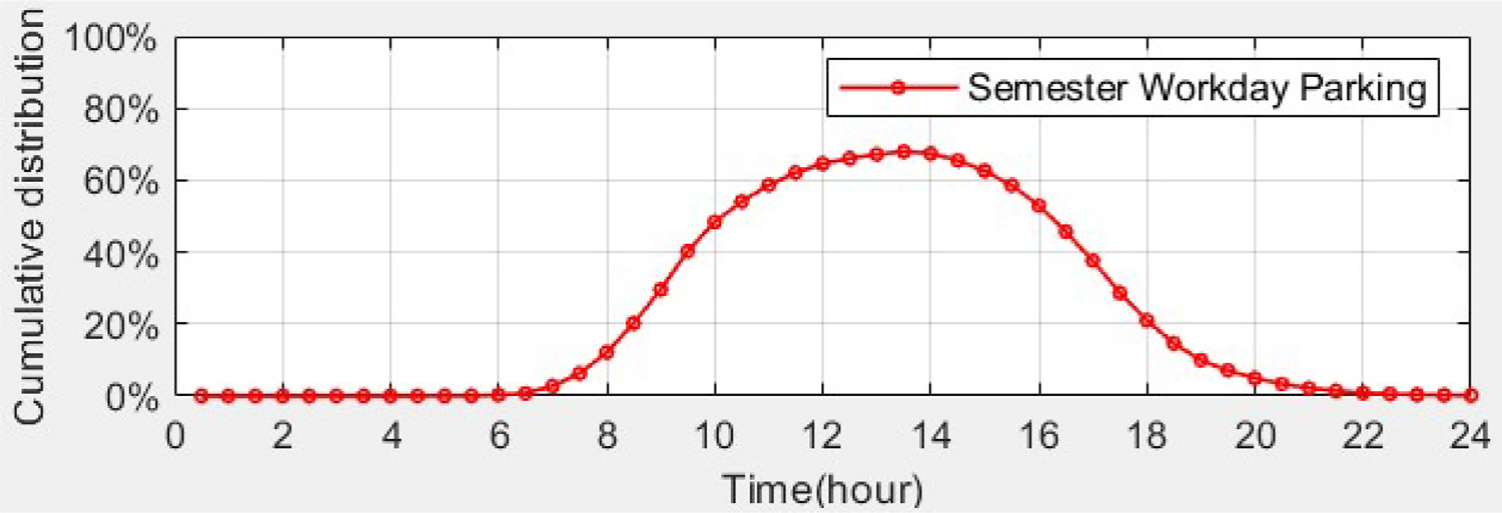
Cumulative distribution of vehicles parked at the Clayton Campus of Monash University.

In Ref. [1], the parking data collected from a real university campus is used to verify the suitability of the distributions extracted from the VISTA data. Fig. 2 presents the annual average cumulative distribution of vehicles parked at the Clayton Campus of Monash University for semester workdays. Comparing Fig. 1(b) with Fig. 2, the cumulative distribution of parked EVs at the workplace as obtained from the VISTA data has a similar pattern and peak percentage to the parking profile of the Clayton Campus for semester workdays. At the Clayton Campus, the highest percentage of vehicle parking also occurs around noon, approximately 68 % of the total parking lots. Also, the annual average cumulative distribution of vehicles parked at the Clayton Campus for non-semester workdays was calculated, and it has a similar pattern to that for semester workdays, with a lower peak (around 26 %). These two real campus parking cumulative distributions can be used to estimate the scaling factor for the number of parked EVs during non-semester workdays and semester workdays.

### 2.6 EV Daily D.csv

*EV Daily D.csv* provides the daily travel distance of each individual EV for a designated number of EVs. This created dataset can be for any number of EVs and any time period (e.g., days, weeks or months). The dataset we have provided contain full-year daily travel distance for 874 EVs. The actual EV charging demand for each day, depending on whether it is a non-semester workday or a semester workday, can be determined using a scaling factor obtained from the vehicle parking data at the Clayton Campus of Monash University, as depicted in Fig. 2. The same distance interval (5 km) is used as was used to obtain the probability distribution. Table 5 provides the description of each column in the datasets.

**Table 5:** Description of datasets: *EV Daily D.csv*.

Column #	Name	Description
1	INDEX_EV	Index representing individual EV
2–366	Day 1–Day 365	Distance data created for each day (in km)

### 2.7 EV Workplace Tar.csv

*EV Workplace Tar.csv* provides the workplace arrival time for each individual EV in the created dataset. The same time interval (30 min) is used as was used to obtain the probability distribution. Table 6 provides the description of each column in the dataset.

**Table 6:** Description of datasets: *EV Workplace Tar.csv*.

Column #	Name	Description
1	INDEX_EV	Index representing individual EV
2–366	Day1–Day 365	Arrival time data created for each day (in minutes, starting from midnight)

### 2.8 EV Workplace Tde.csv

*EV Workplace Tde.csv* provides the workplace departure time for each individual EV in the created dataset. Table 7 provides the description of each column in the dataset.

**Table 7:** Description of datasets: *EV Workplace Tde.csv*.

Column #	Name	Description
1	INDEX_EV	Index representing individual EV
2–366	Day1–Day 365	Departure time data for each day (in minutes, starting from midnight)

## 3 Experimental Design, Materials and Methods

The experimental methods used to process the datasets are implemented using the Microsoft Excel and MATLAB software. These methods for each of the datasets are described below.

### 3.1 Select and Download Raw Data

On the VISTA web platform, there are four publicly available datasets from different collection periods: 2012–20, 2012–18, 2009 and 2007. This paper used the dataset from 2012 to 2018, which is available in a compressed file named *VISTA online data CSV 2012–18*. After downloading and decompressing the entire raw dataset, six CSV files are obtained, which are files created using different sorting criteria, namely, Household, Person, Stop, Trip, Journey to Work, and Journey to Education. Out of the six files, the Trip-based raw data file named *T_VISTA1218_V1.csv* was processed as described in the following 3.2, 3.3, 3.4, 3.5, 3.6 to obtain the dataset required in the research article [1].

### 3.2 Filter Private Vehicle Data

To obtain the travel data of only privately used vehicles, Excel's built-in *filtering* function is used on the raw dataset. Open the raw file *T_VISTA1218_V1.csv*, find LINKMODE in the first row, use the *filter* function to select ‘Vehicle Driver’, and save the file as the filtered dataset. When ‘Vehicle Driver’ is selected then the travel data belonging to only the drivers of privately used vehicles are retained and the data for other types of commuting modes (such as bus, walking, private vehicle passenger, and train) are excluded.

### 3.3 Synthesize Daily Travel Patterns

**Fig. 3:**
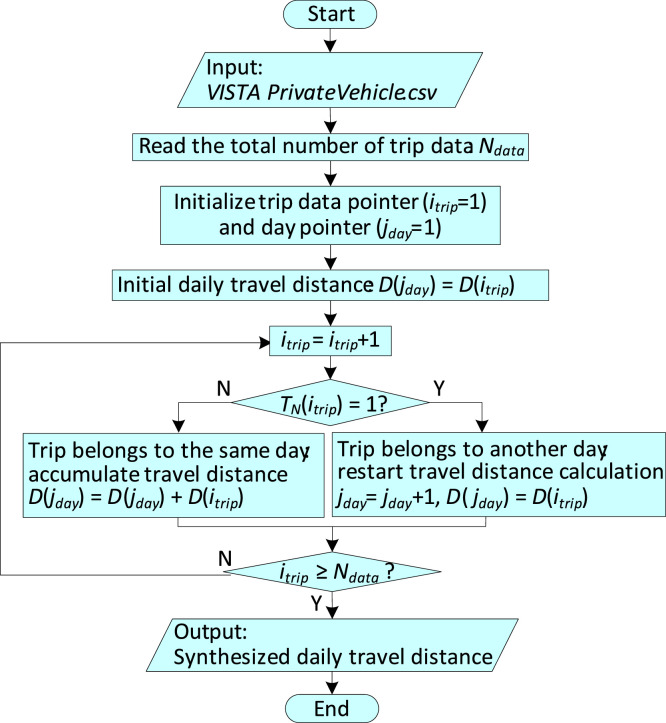
Flowchart of MATLAB data synthesis program for daily travel distance.

**Fig. 4:**
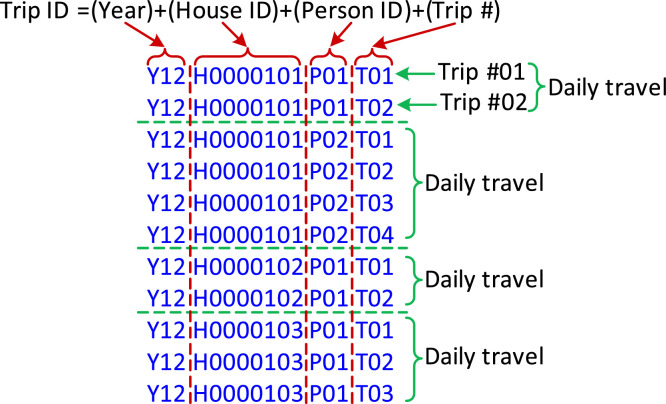
Illustration of the Trip ID from the raw travel dataset *T_VISTA1218_V1.csv*.

The travel pattern used in the original research article [1] includes daily travel distance, and workplace arrival and departure times. To obtain the daily travel distance of each vehicle from the Trip-based dataset, multiple daily trips of the vehicle are synthesized using MATLAB programming. The program flowchart is shown in Fig. 3. Every trip in the raw dataset (*T_VISTA1218_V1.csv*) is identified by a Trip ID which is shown in Fig. 4. It identifies a trip by specifying the year, house ID, person ID making the trip and the trip number. All the trips in a day by the same person are consecutively placed with increasing trip numbers. The trip number (*T_N_*) is a two-digit number and starts counting from one for each day. Using *T_N_* to identify the trips that belong to the same day, the total daily travel distance is obtained by synthesizing the corresponding trip distances. Here, *D* represents the travel distance of one trip.

**Fig. 5:**
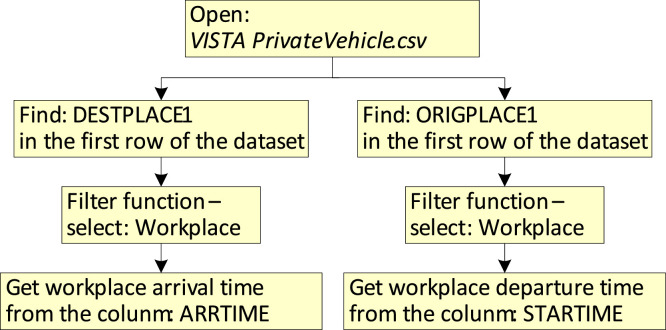
Flowchart of Excel data processing for workplace arrival and departure times.

To obtain workplace arrival and departure times, Excel's built-in *filtering* function is used, and the steps are shown in Fig. 5. Open the *VISTA PrivateVehicle.csv*, find DEPTPLACE1/ORIGPLACE1 in the first row of the dataset and use the *filter* function to select ‘Workplace’, then the trip's arrival and departure times at/from the workplace can be found in the columns ‘ARRTIME’ and ‘STARTTIME’.

Combine the obtained daily travel distances, workplace arrival times and workplace departure times into one dataset and save the file as *VISTA DailyDistance and WorkArrDepTime.csv*.

### 3.4 Extract Probability Distribution

The probability distributions of the daily travel distance, workplace arrival time and workplace departure time are calculated using the MATLAB built-in function ‘ksdensity’. The resulting probability distributions are saved in *VISTA ProbabilityDistribution.csv*. In the original study [1], the distance and time intervals are set to 5 km and 30 min, respectively; these interval values can be set according to requirements.

### 3.5 Calculate Cumulative Distribution

Based on the probability distributions obtained above, the cumulative distributions of workplace arrival and departure times are calculated using (1), which is implemented using MATLAB programming. Here, *CD*(*t*) and *PD*(*t*) represent cumulative distribution and probability distribution respectively in time interval *t*. The cumulative distribution of vehicles arriving in time interval *t* is the sum of all the probability distributions of vehicles arriving prior to time interval *t* as was extracted in the previous section. Likewise, the cumulative distribution of vehicles departing in time interval *t* is the sum of all the probability distributions of vehicles departing prior to time interval *t*. Then, the cumulative distribution of EVs parked at the workplace can be obtained by subtracting the departure cumulative distribution from the arrival cumulative distribution. The cumulative distributions are saved in the file *VISTA CumulativeDistribution.csv*.

(1) $$\left\{ \begin{array}{cc} CD\left(t\right)=PD\left(t\right), & \;if\;t=1\;\\ CD\left(t\right)=CD\left(t\text{-}1\right)+PD\left(t\right), & \;if\;t>1\; \end{array}\right.$$

### 3.6 Create Individual EV Data

**Fig. 6:**
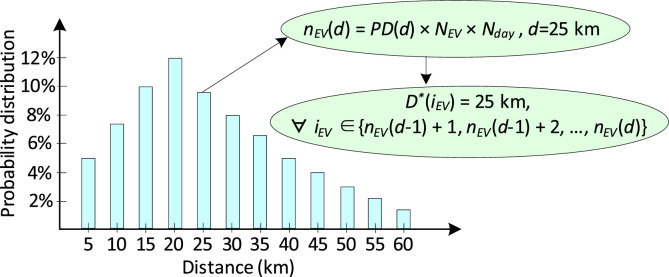
Illustration of the method to obtain the distances travelled daily by individual EVs using the probability distribution of daily travel distance

Fig. 6 aids with the explanation of how a dataset is created for distances travelled daily by individual EVs in a fleet. Here, *N_EV_* represents the designated number of EVs in a fleet and *N_day_* represents the number of days within a designated time period. The data can be created for any number of EVs and time period (e.g., days, weeks or months), depending on the needs of the simulation model. The datasets included with this article are full-year data for 874 EVs, thus, in this study, *N_EV_ =* 874 and *N_day_* = 365. As shown in Fig. 6, using the probability distribution of a certain distance interval (*PD*(*d*)), the number of EVs for this distance (*n_EV_*(*d*)) can be calculated by multiplying the probability distribution *PD*(*d*) with *N_EV_* and *N_day_*. Thus, in the created travel distance dataset D*, the daily travel distance (e.g., *d* = 25 km) is assigned to the *i_EV_*-th EV, here *i_EV_* ∈{*n_EV_*(*d*-1) + 1, *n_EV_*(*d*-1) + 2, …, *n_EV_*(*d*)}. Using the same method, datasets for the daily workplace arrival time (T_ar_*) and departure time (T_de_*) can be created for individual EVs in a fleet. To ensure that the data within one dataset is randomly distributed and the three datasets are mutually independent, the MATLAB function ‘randperm’ is used to randomly permute the three datasets created.

## Limitations

One limitation in Section 3.3 is that the raw dataset (*T_VISTA1218_V1.csv*) does not provide any vehicle ID. Therefore, if the vehicle is driven by more than one person then the synthesized daily travel distance will not truly represent the daily distance driven by a single user. Nonetheless, occurrences of such situations are unlikely to be high in case of privately used vehicles, so the processed data is assumed to be representative of the travel patterns of privately used vehicles.

## Ethics Statement

The authors have read and followed the ethical requirements for publication in Data in Brief and confirming that the current work does not involve human subjects, animal experiments, or any data collected from social media platforms. The authors did not need permission to use the primary data from the Victorian Integrated Survey of Travel & Activity.

## CRediT authorship contribution statement

**Yan Wu:** Conceptualization, Methodology, Investigation, Software, Data curation, Visualization, Writing – original draft, Writing – review & editing. **Syed Mahfuzul Aziz:** Supervision, Conceptualization, Methodology, Visualization, Project administration, Writing – review & editing. **Mohammed H. Haque:** Supervision, Conceptualization, Methodology, Visualization, Writing – review & editing.

## Data Availability

Data for: Techno-economic modelling for energy cost minimisation of a university campus to support electric vehicle charging with photovoltaic capacity optimisation (Original data) (Mendeley Data).
